# bHLH Transcription Factors in Cereal Crops: Diverse Functions in Regulating Growth, Development and Stress Responses

**DOI:** 10.3390/ijms26209915

**Published:** 2025-10-12

**Authors:** Song Song, Nannan Zhang, Xiaowei Fan, Guanfeng Wang

**Affiliations:** 1 College of Life Sciences, Henan Agricultural University, Zhengzhou 450002, China; 2 Postdoctoral Station of Crop Science, Henan Agricultural University, Zhengzhou 450002, China; 3 College of Agronomy and Biotechnology, China Agricultural University, Beijing 100193, China; 4 Sanya Institute of China Agricultural University, Sanya 572024, China

**Keywords:** bHLH transcription factor, cereal crops, growth and development, stress response, molecular breeding

## Abstract

Basic helix-loop-helix (bHLH) transcription factors represent one of the largest transcriptional regulator families in cereal crops such as rice, maize, and wheat. They play crucial and diverse roles in regulating key agronomic traits and essential physiological processes. This review provides a systematic synthesis of the functionally characterized bHLH genes across the three major cereals, offering a comparative perspective on their roles in growth, development, and stress responses. We comprehensively summarize their documented functions, highlighting specific regulators such as *TaPGS1* for grain size, rice ILI subfamily for leaf angle, *OsbHLH004* for seed dormancy and maize “*Ms23-Ms32-bHLH122-bHLH51*” cascade for the anther development. Their conserved and species-specific functions in iron homeostasis (e.g., *IRO2*) and in responses to drought, cold, salinity, and pathogens are also detailed. Additionally, we discuss the underlying molecular mechanisms, including specific binding to E-box/G-box cis-elements, protein dimerization, and integration with hormone signaling pathways. By integrating the current knowledge, this review serves as a consolidated and up-to-date reference that highlights the strategic potential of bHLH transcription factors in molecular breeding programs for improving yield, quality, and stress tolerance in cereals.

## 1. Introduction

Transcription factors are key components in the regulatory network of plant gene expression. They regulate gene transcriptional activity by specifically binding to cis-acting elements in the promoter regions of target genes, thereby influencing plant growth and development, metabolic processes and adaptability to environmental changes. In plants, the basic helix-loop-helix (bHLH) transcription factors constitute one of the largest transcription factor families. For instance, more than 162 bHLH genes have been identified in Arabidopsis thaliana [1,2], while in cereal crops such as Oryza sativa (rice), Zea mays (maize) and Triticum aestivum (wheat), the number of bHLH family members is even more substantial, with 183, 231 and 571 genes identified, respectively [3]. The bHLH transcription factors derive their name from their conserved domain, which endows them with the core abilities to involve in DNA binding and protein interaction, enabling them to play important roles in complex cellular signaling pathways and transcriptional regulatory networks [4,5,6,7]. The multiple roles of bHLH transcription factors in plants cover almost all core biological processes. They are not only key regulators of plant growth and development, participating in various stages from seed germination, plant architecture formation, to floral organ development and grain development, but also crucial regulators in plant responses to abiotic and biotic stresses, playing an important role in plant adaptation to adverse environments [5,8,9,10].

Cereal crops are the foundation of global food security, yet their sustainable production is increasingly threatened by climate change. Rising global temperatures, coupled with a higher frequency of extreme weather events, are projected to significantly reduce the yield of major staple crops, challenging regional and global food systems [11,12]. A critical aspect of this challenge is that crops often face combinations of multiple stresses simultaneously in the field, such as drought, heat, and soil nutrient limitations, creating complex conditions that exceed the resilience of traditional crop varieties [12,13]. Moreover, the genetic homogeneity of global agriculture, which depends on a few major cereal species, increases the risk of large-scale yield losses [14]. In this context, breeding climate-resilient crops has become an urgent goal [15]. Achieving this goal requires a deep understanding of the molecular regulators that help plants cope with these complex environments. As mentioned above, the bHLH family constitutes one of the largest transcription factor families in plants. Given their important roles in both growth and stress responses, they are likely to play a key part in how crops cope with combined environmental stresses. In cereal crops such as rice, maize, and wheat, bHLH proteins regulate many key agronomic traits. Therefore, a comprehensive review of the functional diversity and regulatory mechanisms of bHLH transcription factors is essential, as it provides the fundamental knowledge and genetic resources needed for developing next-generation crops with enhanced resilience and stable yields under a changing climate.

This review aims to sort out the multiple roles of bHLH transcription factors in main cereal crops including rice, maize and wheat, and the transcriptional regulatory mechanisms they function through. By integrating the latest research results, this review seeks to comprehensively present the latest advancements in research on bHLH transcription factors in cereal crops and outline future research directions. This review systematically summarizes the roles of bHLH transcription factors in rice, maize, and wheat, with an emphasis on rice due to its well-established genetic and molecular framework. Where possible, we highlight parallel functions in maize and wheat.

## 2. Structural Characteristics and Regulatory Mechanisms of bHLH Transcription Factors

The bHLH transcription factor family is large and functionally diverse, yet their core functions depend on their conserved bHLH domain. An in-depth understanding of this domain is the basis for analyzing their multiple action mechanisms.

### 2.1. Structural Basis: Sequence Conservation to Functional Specificity

**Figure 1:**
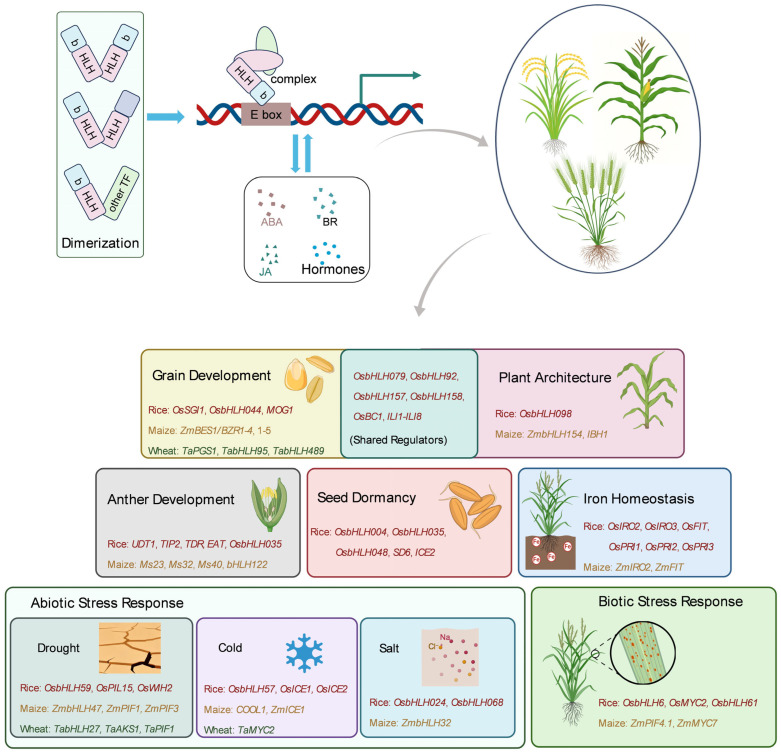
Schematic overview of the molecular mechanisms and diverse functions of bHLH transcription factors in cereal crops. The bHLH transcription factor is characterized by two conserved domains: the basic region, which facilitates DNA binding to E-box motifs (CANNTG), and the helix-loop-helix (HLH) domain, which mediates protein dimerization. bHLH proteins can form homodimers or heterodimers with typical/atypical bHLH proteins or distinct types of transcription factors, thereby vastly expanding their regulatory spectrum and specificity. Their activities are often integrated with hormonal signaling pathways, such as those of ABA, BR, and JA. Functioning as master regulators, bHLH transcription factors orchestrate a wide array of crucial agronomic traits throughout the life cycle of cereal crops, including grain development, plant architecture, anther and pollen formation, iron homeostasis, and responses to abiotic (e.g., drought, cold, salinity) and biotic stresses. Representative functionally characterized genes from rice, maize, and wheat are highlighted for each process.

The bHLH domain typically consists of approximately 60 amino acid residues, comprising a basic region and two helix regions connected by a flexible loop region [16]. The basic region, enriched in basic amino acids (e.g., arginine and lysine), is responsible for specific DNA binding [17,18]. Most bHLH transcription factors recognize and bind to E-box cis-elements in the promoter regions of target genes via the basic region. The core sequence of E-boxes is generally 5′-CANNTG-3′, in which N can be any nucleotide [19]. However, different subfamilies or even individual members within the same subfamily may exhibit distinct binding preferences for E-box variants such as G-box (5′-CACGTG-3′) or non-canonical E-boxes. These differences in sequence recognition specificity constitute a key basis for functional divergence within the bHLH family [4,6,20,21]. The two helix regions, linked by the loop region, form a helix-loop-helix structure that primarily mediates interactions between bHLH proteins, enabling the formation of homodimers or heterodimers [5]. Dimerization represents a core functional mode of bHLH transcription factors. By forming heterodimers, bHLH proteins can interact with other bHLH proteins or transcription factors of different types, significantly expanding their regulatory scope and specificity [7,9,19,21] (Figure 1).

Based on the sequence characteristics and functional relevance of the bHLH domain, the bHLH family can be further divided into multiple subfamilies. In Arabidopsis thaliana, the 162 bHLH members can be clustered into 12 subfamilies, while in gramineous crops such as rice, maize and wheat, researchers have further subdivided the bHLH family into 36 subfamilies by referencing the classification system of Arabidopsis, including 28 cross-species conserved subfamilies and 8 monocot-specific subfamilies [3]. Beyond the conserved bHLH domain, bHLH proteins from different subfamilies often contain additional functional domains, such as the Leucine Zipper (LZ) and Per-Arnt-Sim (PAS) domains [7]. These extra domains further enhance the diversity of protein interactions and may influence subcellular localization, stability, or binding to other signaling components, thereby endowing bHLH proteins with more precise regulatory specificity [22]. Notably, some bHLH proteins lack a complete basic region and are termed “atypical bHLH proteins”. These proteins typically do not bind DNA directly but instead exert inhibitory effects by forming heterodimers with canonical DNA-binding bHLH proteins, thereby modulating the latter’s DNA-binding activity or transcriptional activity [23,24].

### 2.2. Core Transcriptional Regulatory Mechanisms

The regulatory functions of bHLH transcription factors center on their precise control of target gene transcription, which is achieved through multi-faceted molecular mechanisms. Firstly, it’s DNA binding and selection of target genes. bHLH proteins recognize and bind to cis-acting elements, primarily the E-box (CANNTG) and its variants, in the promoters of target genes [4,6,20,21,25]. Variations in binding preferences among different bHLH proteins for E-box variants are key determinants of target gene specificity [4]. Genome-wide mapping of bHLH binding sites using techniques such as ChIP-seq or DAP-seq could reveal their target genes distribution patterns [26,27,28]. DNA binding occurs within the chromatin environment, where bHLH proteins cooperate with histones and may induce DNA release from nucleosomes to access binding sites [22]. During stomatal development in *Arabidopsis*, bHLH factors collaborate with chromatin remodeling complexes (SWI/SNF) and histone acetyltransferases (HAC1) to modulate cis-element accessibility, overcoming repressive chromatin states to activate target genes [29]. Although characterized in model plants, these mechanisms provide foundational insights for cereal crop research. Secondly, it’s bidirectional regulation. A bHLH transcription factor can be a transcriptional activator or a repressor. bHLH factors can function as transcriptional activators by recruiting co-activators or as repressors by recruiting co-repressors or forming inactive heterodimers with atypical bHLH proteins [23,26,27,30]. This dual function allows bHLH proteins to fine-tune gene expression in complex networks. In addition, post-translational modifications (PTMs) dynamically regulate bHLH proteins, modulating their activity, stability, subcellular localization and interaction capabilities [4,5,20,31]. These modifications, which include phosphorylation, ubiquitination and glycosylation, enable bHLH proteins to rapidly respond to environmental changes and developmental signals, facilitating flexible regulation of gene expression [6,32].

## 3. Diverse Roles in Growth, Development and Metabolism Regulation

bHLH transcription factors play extensive and critical regulatory roles throughout the entire lifecycle of cereal crops, from seed germination to grain maturation, and are involved in nearly every developmental stage (Figure 1).

### 3.1. Grain Development and Yield

Grains are the main harvested organs of cereal crops, and their size, weight and quality directly determine crop yield. Numerous studies have demonstrated bHLH transcription factors play pivotal roles in grain development and yield formation of cereal crops (Table 1). In wheat, the bHLH gene *TaPGS1* is specifically expressed in grains at 5–20 days after flowering. Its overexpression in both wheat and rice can significantly increase grain weight and size, and also enhance the contents of carbohydrates and total proteins. *TaPGS1* positively regulates the expression of the seed development-regulating gene *TaFl3* by directly binding to its E-box motif [33]. *TabHLH95* positively regulates starch synthesis in grain of wheat. Knockout mutants show smaller grains and reduced starch content, while overexpression enhances starch accumulation and grain weight. TabHLH95 promotes starch synthesis by binding to the G-box on the promoters of key starch synthesis enzyme genes such as *AGPL1/2* (ADP-glucose pyrophosphorylase) and *ISA1* (isoamylase) to enhance their transcription. TabHLH95 lacks transcriptional activation function, but can interact with the transcription factor TaNF-YB1 to form a complex that coordinately regulates starch synthesis [34]. Another atypical bHLH transcription factor, TabHLH489, has been identified as a negative regulator of grain length [35]. Knockout of *TabHLH489* leads to increases in grain length and weight, whereas overexpression exhibits the opposite effect. TaSnRK1α1, the α catalytic subunit of the plant energy sensor SnRK1, can phosphorylate TabHLH489 to induce its degradation to promote grain development. In addition, TaBZR1, a key transcription factor in the brassinosteroid (BR) signaling pathway, binds to the promoter of *TabHLH489* and represses its expression, thereby promoting grain development [35].

In rice, multiple bHLH transcription factors precisely regulate grain size and yield by modulating cell proliferation, starch metabolism and the BR pathway. OsbHLH044 directly binds to the G-box cis-elements of starch synthesis genes (e.g., OsSSIIa, OsWx and OsFLO2) and seed storage protein genes (GluA1, Globulin1) and activates their transcription [36], increasing the synthesis and accumulation of starch and proteins in the endosperm, reducing grain chalkiness, and thereby improving grain quality and yield [36]. OsbHLH92 participates in the noncanonical BR signaling pathway, directly activating the expression of downstream target genes, *OsBU1* (*OsbHLH172*) and its homologous gene *OsBU1* (*OsbHLH172*), both of which encode atypical bHLH transcription factors [37]. These genes facilitate cell proliferation and growth during grain development, significantly increasing grain weight. OsbHLH92 can enhance yield by at least 10% driven by a panicle-specific promoter [37]. As an atypical bHLH protein, OsBUL1/OsbHLH170 forms a heterotrimer with the typical bHLH proteins, OsBC1 and LO9-711, synergistically promoting the expansion of grain development-related cells and grain size [38]. Notably, *OsbHLH92*, *OsBU1*, *OsBUL1* and *OsBC1* are involved in BR signaling and simultaneously regulate leaf angle [37,38]. Similarly to these genes, *OsbHLH079* also participates in the regulation of grain size and leaf angle via BR signaling [39]. Furthermore, the bHLH protein MOG1 forms a heterodimer with OsbHLH107 to synergistically activate the expression of *LOG* (a key cytokinin-activation enzyme) and *EXPLA1* (a cell expansion gene), thereby promoting young panicle development and significantly increasing both grain number and grain weight. Natural variation in the MOG1 allele (Hap-HNW) with higher transcriptional activity has been identified as a valuable genetic resource for yield improvement in rice breeding [40]. *OsFIF3* (*FLO2 interaction factor 3*) exerts a negative impact on grain quality and yield. It can bind to the G-box motif in the promoters of key starch metabolism genes, inhibiting the expression of *FLO2* (a starch synthesis regulatory gene) and *SUT1* (a sucrose transporter). The repression of these two genes leads to loose arrangement and insufficient filling of starch granules, resulting in increased chalkiness and reduced grain weight in *OsFIF3* overexpression lines [28].

In maize, the bHLH transcription factors ZmBES1/BZR1-5 and ZmBES1/BZR1-4 both positively regulate kernel development and yield [27,41]. The SNPs in *ZmBES1/BZR1-5* are associated with kernel width and kernel weight. Overexpression of *ZmBES1/BZR1-5* increases seed size in Arabidopsis and rice, while the mutants in maize exhibit smaller kernels [27]. The protein of *ZmBES1/BZR1-5* contains bHLH and BAM domains, lacks transcriptional activity as a monomer, and needs to form a homodimer through the BAM domain. It binds to the promoters of *AP2/EREBP* genes and inhibits their transcription [27]. Overexpression of *ZmBES1/BZR1-4* increases kernel size and yield, and mutants have smaller kernels. ZmBES1/BZR1-4 binds to the E-box in the promoters of *ZmMBP1* and *ZmPum6* to activate their transcription, and interacts with ZmTLP5 to enhance the activation activity [41].

### 3.2. Plant Architecture

Plant architecture is an important agronomic trait influencing planting density and light use efficiency in cereal crops, with leaf angle being a key determinant of plant architecture [42]. BR signals directly increase leaf angle by promoting the proliferation and elongation of adaxial cells in the lamina joint [43]. As key regulatory nodes in BR signaling pathway, bHLH transcription factors precisely regulate leaf angle by enhancing or inhibiting BR signaling (Table 1).

*OsBLR1/OsbHLH079/OsBCL2* acts as a positive regulator of BR signaling: its overexpression lines display enhanced BR sensitivity, whereas mutant lines show the opposite phenotype [44]. Additionally, it interacts with OsRACK1A to regulate cell wall-related genes, thereby increasing leaf angle and grain length [44]. OsbHLH98, a negative regulator of BR signaling, is specifically expressed in the adaxial parenchyma of the lamina joint. It can directly bind to the E-Box and G-Box in the promoter region of *OsBUL1/OsbHLH170*, repressing its expression to counteract BR-induced cell elongation, and thereby negatively regulating leaf angle [45].The ILI subfamily of rice bHLH family encodes atypical bHLH transcription factors that lack the basic region, i.e., the DNA-binding domain, and positively regulate leaf angle via the BR signaling pathway [23,24,38,46,47,48]. Several members of the ILI subfamily were detected to be significantly associated with the flag leaf angle by genome-wide association study in a rice germplasm collection, indicating that the natural variations in ILI members have effects on the leaf angle of different rice varieties [49]. Another two atypical bHLH transcription factors, OsbHLH157 and OsbHLH158, which lack transcriptional activation activity, play negative regulatory roles in rice BR signaling [23]. ILIs proteins can interact with OsbHLH157/158 to form heterodimers, which antagonistically regulate BR signals to control traits such as leaf angle, plant height and grain shape [23], and this mechanism is conserved in Arabidopsis and rice [24,50].

The regulation of leaf angle by bHLH transcription factors through BR signaling is often accompanied by alterations in grain size. For example, the aforementioned grain size-regulating genes, such as *OsbHLH079*, *OsbHLH92* and *OsBUL1*, also regulate leaf angle [23,37,38,39]. However, the regulation of plant architecture and grains size by BR is not completely coupled. For instance, mutants and overexpression plants of *OsbHLH98* exhibit no significant changes in the grain size, which may be attributed to its tissue-specific expression pattern [45].

Compared with rice, there are fewer reports on bHLH genes regulating plant architecture in maize and wheat. In maize, ZmbHLH154 directly binds to the promoter of the cell wall-related gene *ZmXTH1* and induces its expression, thereby regulating internode elongation and affecting plant height [51]. Another bHLH transcription factor, ZmIBH1, negatively regulates cell elongation and plant height by forming a heterodimer with ZmbHLH154, which inhibits the activation activity of ZmbHLH154 [51]. In addition, *ZmIBH1* negatively regulate leaf angle, and its regulatory network involves genes related to cell wall, cell development and hormones [26].

**Table 1:** Characterized bHLH family genes regulating grain development and plant architecture.

Gene Name	Gene Accession	Function	Refs.
*TaPGS1*	TraesCS1A02G102400	Increases grain size and weight.	[33]
*TabHLH95*	TraesCS6A03G0268600	Enhances starch accumulation and improves grain size.	[34]
*TabHLH489*	TraesCS2D02G499200	Negatively regulate grain length and weight.	[35]
*OsbHLH044/OsbHLH096*	Os03g0188400	Positively regulates grain quality and salt tolerance; negatively regulate brown planthopper defense.	[36,52]
*OsSGI1/OsbHLH111*	Os04g0489600	Negatively regulate grain size and weight.	[31]
*OsbHLH92*	Os09g0501600	Positively regulate grain size and leaf angle, and negatively regulate plant height via BR signaling.	[37]
*OsBC1*	Os09g0510500		[38]
*ILI1/OsbHLH154*	Os04g0641700		[23,48]
*ILI2/ATAC2*	Os11g0603000		[23]
*ILI3/OsbHLH153/OsBUL2*	Os03g0171700		[23,49]
*ILI4/OsBU1/OsbHLH172*	Os06g0226500		[45,46]
*ILI5/PGL2/OsBUL1/OsbHLH170*	Os02g0747900		[38]
*ILI6/PGL1*	Os03g0171300		[23]
*ILI7/OsbHLH173*	Os10g0404300		[23,49]
*ILI8/OsbHLH174*	Os10g0403800		[23,49]
*MOG1/qRT9*	Os09g0455300	Increases both grain number and grain weight, regulates drought avoidance through controls root length and thickness.	[40,53]
*OsbHLH107*	Os02g0805250	Enhances grain size.	[54]
*OsFIF3*	Os01g0243400	Increases grain chalkiness and decreases grain weight.	[28]
*ZmBES1/BZR1-4*	Zm00001d019757	Positively regulate kernel size and weight but negatively regulate drought resistance.	[41]
*ZmBES1/BZR1-5*	Zm00001d053975	Positively regulate kernel size and weight.	[27]
*OsBLR1/OsbHLH079/OsBCL2*	Os02g0705500	Positively regulate leaf angle and grain length.	[39,44]
*OsbHLH98*	Os03g0797600	Negatively regulates leaf angle	[45]
*OsbHLH157*	Os02g0178700	Negatively regulates leaf angle and grain size through antagonizing ILI proteins.	[23]
*OsbHLH158*	Os06g0653200		[23]
*ZmbHLH154*	Zm00001d013357	Increases plant height	[51]
*ZmIBH1*	Zm00001d001982	Negatively regulates plant height and leaf angle	[26,51]

### 3.3. Anther and Pollen Development

Reproductive development, especially the formation of anther and pollen, is a key step in crop seed setting and yield formation. Anther development is a highly ordered process involving precise differentiation, proliferation, and programmed cell death (PCD) of somatic cell layers (such as the tapetum) and germ cells [55]. bHLH transcription factors have been widely demonstrated to play crucial roles in anther development and pollen formation in cereal crops [56].

Multiple bHLH transcription factors in rice precisely regulate key processes of rice anther development by constructing a multi-level regulatory network (Table 2). The entire process of anther development, from stamen primordium formation to mature pollen release, is divided into 14 stages [56]. *UDT1* encodes a typical bHLH transcription factor. As an early core factor, *UDT1* is highly expressed in the tapetum and meiotic cells during early anther development (stages 6–8b) [57]. It not only initiates tapetal cell maturation and secondary wall cell differentiation but also indirectly regulates the initiation of tapetal PCD by activating downstream genes such as *TDR* (*OsbHLH5*) [58]. Additionally, it upregulates genes like *OsC6* and *OsCYP703A3* involved in pollen wall precursor synthesis [59]. Mutations in *UDT1* lead to premature tapetal degeneration, abnormal meiosis, and ultimately male sterility [57]. *TIP2* (*OsbHLH141*) functions from the initiation of meiosis to the microspore development stage (stages 6–10), controlling the differentiation of the inner three anther wall layers (tapetum, middle layer, and endothecium) [60]. It activates the expression of *TDR* and *EAT1* by directly binding to their promoters [60,61]. Mutations in *TIP2* result in blocked differentiation of these cell layers, failed tapetal PCD, and inability of microspore mother cells to mature [60,62]. *TDR* dominates the timing of tapetal PCD during stages 7–8b [63,64]. It directly regulates target genes such as *OsCP1* (initiating degradation), *OsC6* (lipid transport) and *OsADF* (protein degradation) by binding to E-box [63,64]. Mutations in *TDR* cause delayed degeneration of the tapetum and middle layer, microspore collapse, and pollen exine defects [63]. *EAT1* (*DTD*, *OsbHLH141*) is expressed in a biphasic pattern (stages 7 and 9–12) [65]. It initiates tapetal PCD by activating *OsAP25* and *OsAP37*, regulates *OsLTPL94* to participate in pollen exine assembly, and collaborates with UDT1 to activate the transcription of 24-nt phasiRNA precursors to maintain meiotic synchrony [65,66]. Mutations in *EAT1* lead to delayed meiosis and abnormal pollen exine [65]. In addition, *OsbHLH035* is expressed at stage 6 and negatively regulated by *OsGRF11* [67]. Overexpression of *OsbHLH035* causes anther curvature and pollen vacuolation [67]. Overall, bHLH transcription factors in rice form a core regulatory cascade “*UDT1-TIP2-TDR/EAT1*”, precisely regulating tapetal development and degradation, pollen wall synthesis, and meiotic processes, ultimately ensuring normal pollen development and fertility [56].

Similarly, in maize, several bHLH transcription factors play key roles in crucial processes like tapetum specialization, cell division and differentiation, pollen wall formation, and tapetal PCD by forming a hierarchical regulatory network (Table 2). These bHLH transcription factor genes include *MS23*, *MS32*, *Ms40* (*bHLH51*) and *bHLH122* [68]. Among them, *MS23* acts as an early dominant factor. It is specifically expressed in secondary parietal cells and differentiated tapetum [69,70]. By inhibiting extra periclinal division of tapetal precursor cells and promoting their differentiation, it ensures that tapetal cells have characteristics such as dense cytoplasm. The mutant *ms23-ref* causes abnormal division of tapetal precursor cells, forming a multi-layered tapetal structure and eventually leading to pollen abortion [68,69]. *MS32* is homologous to rice *UDT1*. It is widely expressed in anthers before meiosis and restricted to the tapetum in the later stage. Its function is to limit periclinal division of tapetal cells and maintain their differentiated state. In the *ms32* mutant, tapetal precursor cells divide excessively and expand, disrupting the development of pollen mother cells [71]. Moreover, *MS32* works with *MAC1* to maintain the integrity of anther structure by inhibiting the disorderly proliferation of somatic cells [71]. Zm*bHLH51* (*Ms40*), as a late regulatory factor, is homologous to sorghum *TDR*. It initiates tapetal PCD by directly regulating genes such as cysteine protease gene *OsCP1* and aspartic protease gene *OsAP25/37*. It also participates in the expression regulation of lipid transfer protein gene *OsC6* and sporopollenin synthesis genes like *OsPKS1/2*. Its mutant *ms40* exhibits delayed degradation of the tapetum and defects in the pollen wall, resulting in non-functional pollen [72]. In addition, bHLH122, which acts downstream of MS23, forms a heterodimer with MS32, and coordinately regulates the maintenance of tapetal function and the expression of genes related to pollen maturation [68].These bHLH factors form a regulatory network through cascade interactions as follows: MS23 acts upstream to regulate bHLH51 and bHLH122; MS32 independently regulates late differentiation; and MS23 forms heterodimers with bHLH51, while MS32 forms heterodimers with bHLH122, respectively, to activate downstream target genes [68]. They jointly coordinate tapetal cell fate determination, nutrient supply, and pollen wall construction to ensure normal pollen development. Mutations in any one of them could result in male sterility.

**Table 2:** Characterized bHLH family genes regulating anther development.

Gene Name	Gene Accession	Function	Refs.
*UDT1/OsbHLH164*	Os07g0549600	Promote the PCD process of tapetal cells, and regulate tapetum development and pollen formation.	[57,58]
*TIP2/OsbHLH141*	Os01g0293100		[60,61]
*TDR/OsbHLH005*	Os02g0120500		[63,64]
*EAT1/DTD/OsbHLH141*	Os04g0599300		[65,66]
*OsbHLH035*	Os01g0159800	Regulates anther development	[67]
*MS23*	Zm00001d008174	Controls tapetum specification and maturation	[68,69]
*MS32*	Zm00001d006565		[68,71]
*M* *s* *40/* *bHLH* *51*	Zm00001d053895	Promote the PCD process of tapetal cells and pollen development.	[72]
*bHLH122*	Zm00001d017724	Regulates tapetal function and pollen development.	[68]

### 3.4. Seed Dormancy and Germination

The dormancy and germination of cereal crops are crucial for their survival, reproduction and agricultural production. Appropriate dormancy characteristics can reduce pre-harvest sprouting in the field, and synchronized germination facilitates agricultural management [73]. Seed dormancy and germination are regulated by various environmental factors and endogenous signals, among which abscisic acid (ABA) and gibberellins (GAs) are key regulatory hormones [74]. bHLH transcription factors play a core role in regulating seed dormancy and germination mainly by mediating hormone-related pathways [75,76,77]. At present, research on the regulation of seed dormancy and germination by bHLH is mainly focused on rice (Table 3), with relatively few reports on maize and wheat [78].

In rice, *OsbHLH004* is a key repressor of seed dormancy, and its mutation enhances seed dormancy [76]. OsbHLH004 can directly bind to the promoters of ABA synthesis gene *OsNCED3* and GA metabolism gene *OsGA2ox6* to inhibit their expression, thus inhibiting ABA synthesis and promoting GA degradation. In addition, phosphatidylethanolamine-binding proteins OsMFT1/2 interacts with OsbHLH004 and alleviates the binding of OsbHLH004 to *OsNCED3* and *OsGA2ox6*, dynamically regulating dormancy during seed maturation [76]. Another two bHLH transcription factors, SD6 and ICE2, regulate rice seed dormancy and germination through antagonistic effects [77]. As a repressor of dormancy, SD6 binds to the G-box motif of ABA catabolism gene *ABA8OX3* and activates its expression, reducing ABA content to promote germination. By contrast, ICE2 binds to the E-box motif of *ABA8OX3* and inhibits its expression, increasing ABA accumulation to maintain dormancy. Another bHLH gene, *OsbHLH048*, is activated by SD6 and inhibited by ICE2, and then inhibits the expression of ABA synthesis gene *NCED2*, forming the *SD6/ICE2*-*OsbHLH048*-*NCED2* cascade pathway. Furthermore, by editing SD6 in rice and its homologous gene in wheat, respectively, the occurrence of pre-harvest sprouting can be reduced. Therefore, SD6 is a useful breeding target for alleviating pre-harvest sprouting in cereal crops [77].

**Table 3:** Characterized bHLH family genes regulating seed dormancy and germination.

Gene Name	Gene Accession	Function	Refs.
*OsbHLH004*	Os10g0544200	Represses seed dormancy through inhibiting ABA synthesis and GA degradation.	[76]
*SD6*	Os06g0164400	Negatively regulate seed dormancy.	[77]
*ICE2*	Os01g0928000	Positively regulate seed dormancy.	[77]
*OsbHLH048*	Os02g0759000	Negatively regulate seed dormancy through ABA biosynthesis.	[77]
*OsbHLH035*	Os01g0159800	Promote seed germination and salt tolerance.	[79]

### 3.5. Iron Homeostasis

Iron plays a crucial role in many important physiological processes such as photosynthesis, respiration, and nitrogen metabolism [80,81]. Although abundant in the Earth’s crust, iron has greatly limited bioavailability in aerobic environments and alkaline soils, often leading to iron deficiency stress in plants. Maintaining iron homeostasis in cereal crops is thus of great significance for ensuring crop yield and quality [80]. Plants employ two main strategies to acquire iron: Strategy I and Strategy II [82,83,84]. Strategy I is mainly found in dicotyledonous plants and non-graminaceous monocotyledonous plants. When plants perceive an iron deficiency signal, iron reductases on the root plasma membrane reduce Fe(III) to Fe(II), which is then transported into cells via iron transporters [85]. Strategy II is unique to gramineous plants. Under iron deficiency conditions, plants synthesize and secrete phytosiderophores, such as mugineic acids, which can efficiently chelate Fe(III) in the soil to form Fe(III)–phytosiderophore complexes [86]. These complexes are then transported into root cells via specific transporters. In cereal crops, multiple bHLH transcription factors form a complex and precise molecular network to regulate iron homeostasis through Strategy II [82]. The core mechanism involves activation, auxiliary regulation and negative feedback balance, exhibiting both conservation and specificity across different species.

In rice, *OsIRO2*(*OsbHLH056*) serves as a core activator of iron acquisition [87,88]. Under iron deficiency conditions, it is strongly induced and activates genes involved in phytosiderophore synthesis (e.g., *OsNAS1/2*, *OsNAAT1*, *OsDMAS1*) and transport (e.g., *OsYSL15*, *TOM1*) [88]. OsFIT (OsbHLH156) regulates the subcellular localization of OsIRO2 through physical interaction [89,90]. Under normal conditions, OsIRO2 is mainly distributed in the cytoplasm, while under iron deficiency, OsFIT forms a heterodimer with OsIRO2, promoting its nuclear translocation and thereby activating downstream genes. Knockout of *OsFIT* leads to leaf chlorosis and reduced iron content in rice under iron deficiency, and blocks the induced expression of Strategy II-related genes (e.g., *OsNAS2* and *OsYSL15*) [90]. This indicates that OsFIT is an essential cofactor for OsIRO2 function, and they together constitute the core activation module of Strategy II. In contrast to the activating role of *OsIRO2*, *OsIRO3* (also a bHLH gene) maintains iron homeostasis through negative regulation [91,92,93]. *OsIRO3* expression is upregulated under iron deficiency, and it suppresses the expression of *OsIRO2* and downstream genes like *OsNAS3* and *OsYSL15* to prevent oxidative damage caused by excessive iron uptake. Its regulatory mechanism may rely on the EAR motif (LxLxL) at the C-terminus, which recruits TOPLESS family repressors to inhibit target gene transcription [92]. Knockout of *OsIRO3* results in excessive iron accumulation and leaf necrosis in rice, confirming its negative regulatory role [93]. Rice iron homeostasis involves multiple other bHLH factors, including the positive regulators *OsbHLH060* (*OsPRI1*), *OsbHLH058* (*OsPRI2*), *OsbHLH059* (*OsPRI3*) and *OsbHLH133*, as well as the negative regulator *OsbHLH061*. *OsPRI1*, *OsPRI2* and *OsPRI3* are induced under iron deficiency, and enhance iron uptake efficiency by activating *OsIRO2* and *OsIRO3* [94,95,96]. OsbHLH061 interacts with PRI1 to form a complex that recruits TOPLESS/TOPLESS-RELATED (TPL/TPR) repressors, thereby suppressing the downstream genes *OsIRO2* and *OsIRO3*. This mechanism negatively regulates the long-distance transport of iron, ensuring a balanced distribution of iron in rice [97]. Another gene *OsbHLH133*, regulates the distribution of iron between roots and shoots, possibly by negatively regulating the translocation of iron from roots to shoots [98].

Studies on the molecular mechanisms of iron homeostasis in maize and wheat are relatively limited (Table 4). Similarly to rice, a pair of bHLH transcription factors in maize, ZmFIT and ZmIRO2, interact to form a heterodimer and activate genes involved in phytosiderophore synthesis and transport [99]. However, the specific regulatory details and networks remain unclear. Transcriptomic analysis of wheat has shown that iron deficiency induces the expression of several bHLH genes (such as *TaIRO2*, homologous to *OsIRO2*), suggesting potential conservation of the regulatory framework with rice [100]. Overall, the bHLH transcription factors in cereal crops maintain iron homeostasis through a hierarchical network of “activation-assistance-inhibition”, providing key targets for breeding iron-deficiency tolerant crops and improving their nutritional quality.

## 4. Roles and Regulatory Networks in Stress Responses

Cereal crops often encounter various stressors, including drought, salinity, extreme temperatures, pests, and pathogens. bHLH transcription factors play extensive and important roles in responses to these stresses and improving stress tolerance.

### 4.1. Adaptation to Abiotic Stresses

Abiotic stresses are major limiting factors affecting the yield and distribution range of cereal crops [101]. bHLH transcription factors are involved in plant adaptation to adverse environments by regulating the expression of stress-responsive genes (Table 5).

#### 4.1.1. Drought

bHLH transcription factors serve as critical nodes in the drought-responsive regulatory networks of cereal crops. They not only activate or repress drought resistance genes but also balance plant growth and stress adaptation through interactions with other proteins and responses to hormonal signals, thus acting as crucial molecular switches linking environmental signals to physiological responses [25].

In rice, bHLH transcription factors regulate the balance between drought response and growth through diverse mechanisms. *OsbHLH59* generates two functionally distinct transcripts via alternative splicing [102]. *OsbHLH59.1* is constitutively expressed under normal conditions, promoting cell division by activating developmental genes such as *TUD1* to enhance plant height and root length. Expression of *OsbHLH59.2* increases with stress duration, activating drought-resistance genes like *OsNCED4* to increase the content of osmotic adjustment substances and antioxidant enzyme activity, thereby regulating drought tolerance. These two transcripts can form heterodimers, with OsbHLH59.1 dominating growth and development while OsbHLH59.2 exerts antagonistic and competitive regulation. The two transcripts undergo stress-dependent splicing switching via the ABA signaling pathway. Thus, OsbHLH59 balances growth and drought response through alternative splicing [102]. OsPIL15, a phytochrome-interacting factor, binds to the PBE-box motif in the promoter of *OsABI5* (a key bZIP transcription factor in the ABA signaling pathway) to promote its expression and induce stomatal closure, thereby negatively regulating transpiration to enhance drought resistance [103]. Additionally, OsPIL15 interacts with OsHHO3 to enhance the regulation of *OsABI5* [103]. *OsbHLH120*, cloned from an upland rice introgression line, controls root length and thickness, which provides a theoretical basis and genetic resource for breeding drought-resistant rice varieties [53].

In maize, the drought response mediated by bHLH transcription factors involves both positive and negative regulators. ZmbHLH47 binds to the E-box element and activates the expression of *ZmSnRK2.9*, which encodes class III SnRK2 kinase [104]. ZmSnRK2.9 phosphorylates downstream transcription factors such as ABF, enhancing the expression of ABA-responsive genes (e.g., *ZmRAB18* and *ZmABF2*), which further increases sensitivity to ABA, reduces electrolyte leakage and MDA accumulation under drought stress, and ultimately improves drought resistance. *ZmPIF3*, a homolog of *OsPIL15*, conserves drought resistant functions by regulating stomatal movement via the ABA signaling pathway [105]. ZmbHLH124 directly binds to the E-box in the promoter of the core drought resistant gene *ZmDREB2A* to activate its expression, further regulating downstream drought responsive genes and enhancing the osmotic adjustment capacity in maize [106]. *ZmPTF1* enhances maize drought resistance through direct regulation of ABA synthesis and root development [107]. On one hand, ZmPTF1 activates *NCED* genes by binding to their G-box elements, increasing endogenous ABA content to activate antioxidant enzymes (POD, SOD) and reduce membrane damage. On the other hand, ZmPTF1 upregulates auxin signaling genes, promotes lateral root primordium formation and elongation to enhance the water uptake capacity of roots. ZmPTF1 also forms a hierarchical regulatory network by activating transcription factors such as NAC30 and CBF4, expanding the expression range of drought-responsive genes [107]. *ZmbHLH137* improves drought tolerance by enhancing the antioxidant system, as it transcriptionally activates antioxidant-related genes involved in glutathione metabolism and flavonoid biosynthesis pathways [108]. The *BES1/BZR1* family genes in maize negatively regulate drought resistance. *BES1/BZR1-1* reduces drought resistance by inhibiting the activation of the antioxidant system [109]. *ZmBES1/BZR1-4,* a key regulator balancing kernel development and drought resistance in maize, negatively regulates drought resistance but positively regulates seed size [41]. ZmBES1/BZR1-4 interacts with the thaumatin-like protein ZmTLP5, and together they bind to the E-box elements in the promoters of *ZmMBP1* and Zm*Pum6* to activate their expression. *ZmPum6* negatively regulates drought resistance by increasing stomatal opening and water loss while *ZmMBP1* promotes seed development [41].

Wheat also has several bHLH transcription factors regulating drought resistance. *TabHLH27* is a key regulator that mediates drought responses through multiple molecular mechanisms, balancing plant growth and drought resistance [110]. Under short-term drought stress, *TabHLH27* is rapidly induced and activates stress related genes such as *TaCBL8* and represses growth related genes. TabHLH27 can also form complexes with transcription factors such as TaABI3 and TabZIP62 to coordinately bind to G-box/E-box elements and regulate downstream genes [110]. TaAKS1, also a bHLH transcription factor, interacts with TaERF87 to synergistically activate proline synthesis genes such as *TaP5CS1* [111]. Overexpression of *TaAKS1* exhibits increased proline content and drought survival rates [111]. *TabHLH1* responds to ABA signals and upregulates the expression of ABA receptor and kinase genes, thereby promoting stomatal closure and increasing the accumulation of osmotic substances [112]. TabHLH49 directly binds to the G-box element in the promoter of the dehydrin protein gene *WZY2*, promoting its expression and reducing membrane damage to enhance drought resistance [113]. TaPIF1 induces stomatal closure by binding the E-box elements of genes like *TaABI5* to reduce water loss, and synergizes with *TaABI5* and *TaAKS1* to enhance the expression of proline synthesis and ABA-responsive genes [114].

In summary, bHLH transcription factors in rice, maize and wheat share common regulatory patterns. They regulate downstream genes by binding to elements such as E-box and G-box to balance growth and stress adaptation; they often interact with other proteins to form regulatory networks and integrate ABA signals. Ultimately, they enhance drought resistance by regulating osmotic adjustment, antioxidant processes, stomatal movement, and root development, thus functioning as key nodes that link environmental signals to physiological responses.

#### 4.1.2. Cold Stress

Cold stress severely impairs the growth and yield of cereal crops. Some bHLH transcription factors are involved in regulating cold tolerance. In rice, *OsbHLH57* functions as a key positive regulator [115]. It promotes trehalose accumulation by upregulating trehalose synthesis genes *TPS1* and *TPP1*, and enhances the activity of antioxidant enzymes like POD and SOD to reduce ROS accumulation. It also upregulates transcription factors including DREB1A/B, thereby activating the expression of CBF signaling genes. Additionally, OsbHLH57 improves grain yield by optimizing seed setting rate and grain size, simultaneously improving cold resistance and yield [115]. OsICE1 and OsICE2, homologs of Arabidopsis ICE1, are MYC-like bHLH transcription factors. They synergistically regulate cold responses by activating CBF/DREB1 genes and interacting with OsMYBS3. Overexpression of *OsICE1/OsICE2* in Arabidopsis significantly enhances cold tolerance and increases survival rates [116]. In maize, *COOL1* (*ZmbHLH76*) acts as a critical negative regulator of cold stress responses [117]. It binds to the G-box elements to repress the expression of core cold responsive gene *DREB1* and trehalose synthesis gene *TPS*, thereby weakening the cold tolerance. Simultaneously, cold-activated kinase CPK17 phosphorylates COOL1 to enhance its stability, further suppressing the cold responses. Additionally, natural variations in its promoter affect the binding capacity of the upstream bZIP transcription factor HY5, leading to variation in cold tolerance [117]. Another positive regulator, *ZmICE1*, also encodes a bHLH transcription factor [118]. It reduces Glu/Asn synthesis to avoid mitochondrial ROS bursts and directly activates *DREB1s*. Variations in its promoter contribute to variations in cold tolerance among maize inbred lines [118]. In wheat, *TaMYC2* is induced by extremely low temperatures. It interacts with TaICE41 and TaJAZ7 to activate the CBF signaling pathway, strengthening cold responsive signals [119].

bHLH transcription factors in rice, maize, and wheat precisely regulate cold responses through the conserved ICE-CBF-COR core pathway, combined with metabolic regulation, including trehalose and proline synthesis, ROS scavenging, and protein interactions with other transcription factors. Natural variations enable crops to adapt to cold environments at different latitudes, providing key targets and theoretical basis for genetic improvement of cold tolerance in cereal crops.

#### 4.1.3. Salt Stress

In terms of salt stress responses, rice *OsbHLH035* is involved in regulating seed germination and seedling recovery under salt stress [79]. It maintains ion balance by regulating ABA metabolism and the expression of ion transporters *OsHKTs* [79]. *OsbHLH024* functions as a negative regulator of salt tolerance. It represses the expression of ion transporter genes including *OsHKT1;3*, *OsHAK7* and *OsSOS1* [120]. Mutants of *OsbHLH024* exhibit enhanced salt tolerance with higher antioxidant enzyme activity, lower ROS and MDA levels, and better ion balance [120]. In addition to regulating grain quality, *OsbHLH044* acts as a positive regulator in salt stress responses. It directly binds to the G-box elements in the promoters of target genes to activate ion balance-related genes (e.g., *OsHKT1;3*, *OsHAK7*, *OsSOS1*, *OsNHX2*) and the ABA-responsive gene *OsLEA3*, thereby reducing Na^+^ accumulation and maintaining K^+^ balance [120]. *OsbHLH068* shares partial functional redundancy with Arabidopsis *AtbHLH112*, enhancing salt tolerance by reducing ROS accumulation under salt stress and promoting root elongation [121]. In maize, ZmbHLH32 regulates salt tolerance through the ZmIAA9-ZmARF1 module: ZmbHLH32 binds to the ZmIAA9 promoter to activate its expression, and ZmIAA9 interacts with ZmARF1 to repress the expression of ROS-scavenging genes [46]. *ZmbHLH55* enhances salt tolerance by regulating ascorbic acid synthesis to increase the activity of plant antioxidant enzymes [122]. Recently, in wheat, a comprehensive study revealed that *TabHLH319* confers salt tolerance by significantly enhancing the activities of antioxidant enzymes and the accumulation of proline, thereby reducing oxidative damage [123]. These studies demonstrate that bHLH transcription factors counteract salt stress by regulating ion balance, osmotic adjustment, and antioxidant systems.

**Table 5:** Characterized bHLH family genes regulating abiotic stress response.

Pathway	Gene Name	Gene Accession	Function	Refs.
Drought	*OsbHLH59*	Os02g0116600	Generates two functionally differentiated transcripts to balance drought response and growth.	[102]
	*OsPIL15*	Os01g0286100	Promote stomatal closure and enhance drought resistance; negatively regulate grain size and weight.	[103,124]
	*OsWIH2*	Os03g0431100	Improve drought tolerance by regulating wax biosynthesis and reducing ROS accumulation.	[125]
	*ZmbHLH47*	Zm00001d048901	Reduces electrolyte leakage and MDA accumulation and improves drought resistance.	[104]
	*ZmPIF3*	Zm00001d008205	Promote stomatal closure and enhance drought resistance.	[105]
	*ZmbHLH124*	Zm00001d037749	Enhances the osmotic adjustment capacity and improves drought resistance.	[106]
	*ZmPTF1*	Zm00001d045046	Enhances maize drought resistance through regulation of ABA synthesis and root development.	[107]
	*ZmbHLH137*	Zm00001d033848	Improves drought tolerance by enhancing the antioxidant system	[108]
	*TabHLH27*	TraesCS2A02G271700	Orchestrates root growth and drought tolerance.	[110]
	*TaAKS1*	TraesCS7A02G435800	Increases proline content and improves drought tolerance.	[111]
	*TabHLH1*	TraesCS7A02G157700	Improves drought tolerance by promoting stomatal closure and increasing osmotic substance accumulation.	[112]
	*TabHLH49*	TraesCs7A02G211900	Enhances drought resistance.	[113]
	*TaPIF1*	TraesCS1A02G083000	Improves drought tolerance by promoting stomatal closure and enhancing proline synthesis.	[114]
Cold	*OsbHLH57*	Os07g0543000	Improves chilling tolerance.	[115]
	*OsICE1*	Os11g0523700	Positively regulate cold tolerance.	[116]
	*OsICE2*	Os01g0928000	Positively regulate cold tolerance. Enhances cold tolerance.	[116]
	*COOL1/ZmbHLH76*	Zm00001d040148		[117]
	*ZmICE1*	Zm00001d042263	Enhances cold tolerance through repressing mitochondrial ROS bursts.	[118]
	*TaMYC2*	TraesCS1A02G193200, TraesCS1B02G208000 and TraesCS1D02G196900	Improves freezing tolerance.	[119]
Salt	*OsbHLH024*	Os01g0575200	Negatively regulate salt tolerance.	[120]
	*OsbHLH068*	Os04g0631600	Enhances salt tolerance.	[121]
	*ZmbHLH32*	Zm00001d004007	Enhances salt tolerance.	[126]

### 4.2. Biotic Stress Defense

Infections by pathogens and pests represent another major type of stress affecting the yield and quality of cereal crops. bHLH transcription factors are also involved in plant defense responses to pathogenic stresses, usually interacting with other signaling pathways such as those of jasmonic acid (JA) and salicylic acid (SA).

In the disease resistance processes of cereal crops, bHLH transcription factors play core roles via multiple mechanisms, including the regulation of hormonal signaling pathways, phytoalexin biosynthesis, defense gene expression, and protein–protein interactions. Their specific functions and mechanisms across different crops exhibit both commonalities and specificity.

In rice, multiple bHLH transcription factors regulate immunity signals to achieve disease resistance (Table 6). OsbHLH6 dynamically balances SA and JA signals through nucleo-cytosolic trafficking [127]. When localized in the nucleus, OsbHLH6 activates JA-responsive genes and represses SA signaling genes, negatively regulating resistance to *Magnaporthe oryzae* (rice blast fungus). Upon rice blast infection, the SA regulator OsNPR1 sequesters OsbHLH6 in the cytoplasm, relieving the repression of SA signaling and initiating immune responses [127]. The atypical bHLH transcription factor OsHLH46 positively regulates rice blast resistance by competitively binding to OsbHLH6, preventing the formation of OsbHLH6 homodimer that binds downstream genes [128]. *OsbHLH5* enhances resistance to *Xanthomonas oryzae pv. oryzae* by promoting the accumulation of phenolamides and diterpenoid phytoalexins [129]. OsbHLH057 binds to the AATCA element in the promoter of the defense gene *Os2H16*, upregulating defense genes such as *PR1* and *PR10*, thereby enhancing resistance to sheath blight, bacterial blight, and drought tolerance [130]. OsbHLH034 is induced by JA and interacts with OsJAZ9 to upregulate secretory peroxidase genes. This upregulation increases lignin accumulation and thereby enhances resistance to bacterial blight, though it is accompanied by higher sensitivity to salt stress [131]. *OsMYC2* participates in the immune responses against diverse pests and pathogens including brown planthoppers, rice stripe virus, rice sheath blight, and rice blast, via the JA signaling pathway [132,133,134,135]. Additionally, the atypical bHLH gene *OsHLH61* is induced by JA and repressed by SA. OsHLH61 forms a heterodimer with OsbHLH96 and influences brown planthopper resistance by regulating PR genes. OsbHLH96 may also interact with OsJAZ3 to participate in JA-SA signal crosstalk [52].

In maize, *ZmPIF4.1* acts as a negative regulator of resistance to Gibberella stalk rot (caused by *Fusarium graminearum*) via the ZmPIF4.1-ZmPTI1c-ZmMYB31 module [136]. Specifically, ZmPIF4.1 directly binds to the G-box in the promoter of the receptor-like cytoplasmic kinase gene *ZmPTI1c* to activate its expression. Subsequently, ZmPTI1c phosphorylates ZmMYB31 (a MYB transcription factor) to repress lignin synthesis-related genes, thereby weakening maize resistance to Gibberella stalk rot [136]. ZmMYC7, a putative MYC2 ortholog, can interact with JAZ family members in maize [137]. Upon pathogen infection and JA signal activation, JAZ proteins are degraded, releasing ZmMYC7 to initiate downstream disease resistance pathways. ZmMYC7 directly binds to the G-box cis-element in the ZmERF147 promoter to activate its expression, further regulating pathogenesis-related genes and positively enhancing maize resistance to *Fusarium graminearum* [137].

**Table 6:** Characterized bHLH family genes regulating biotic stress response.

Gene Name	Gene Accession	Function	Reference
*OsbHLH6*	Os04g0301500	Regulates resistance to *Magnaporthe oryzae*	[127,128]
*OsHLH46*	Os01g0108600	Enhances disease resistance to *Magnaporthe oryzae*	[128]
*OsbHLH5*	Os01g0195801	Enhances resistance to Xanthomonas oryzae.	[129]
*OsbHLH057*	Os07g0543000	Enhances resistance to sheath blight, bacterial blight, and drought tolerance	[130]
*OsbHLH034*	Os02g0726700	Enhances resistance against rice bacterial blight.	[131]
*OsMYC2*	Os10g0575000	Enhances resistance against rice brown planthoppers, stripe virus, sheath blight, and rice blast.	[132,133,134,135]
*OsHLH61*	Os07g0676600	Positively regulate defense to brown planthoppers.	[52]
*ZmPIF4.1*	Zm00001d031044	Negatively regulate immunity to gibberella stalk rot.	[136]
*ZmMYC7*	Zm00001d030028	Increase resistance to *Fusarium graminearum*.	[137]

## 5. Conclusions and Perspectives

As a large and functionally diverse family of transcriptional regulators in cereal crops, bHLH transcription factors mediate DNA binding and protein interaction through the conserved bHLH domain, constructing elaborate regulatory networks in growth, development, metabolic balance and stress response (Figure 1). In terms of growth and development, from grain formation, plant architecture establishment, and anther development, to seed dormancy, the bHLH family achieves precise regulation of key agronomic traits in crops by binding to cis-elements such as E-box/G-box, forming heterodimers and responding to hormone signals. In stress adaptation, the function of these bHLH transcription factors exhibits a “balance” characteristic; by integrating signals such as ABA, JA, and SA, they regulate osmotic adjustment, reactive oxygen species (ROS) scavenging, and stress responsive gene expression, ultimately enhancing crop resistance to drought, cold, salt stress and diseases.

This review has synthesized the current understanding of bHLH regulators in the three major cereals: rice, maize, and wheat. It is evident, however, that the depth of functional characterization is not evenly distributed. Rice, as a model cereal, provides the most comprehensive insights, with well-defined regulatory cascades such as the “UDT1-TIP2-TDR/EAT1” module in anther development and the “SD6/ICE2-OsbHLH048” pathway in seed dormancy. In contrast, while maize and wheat possess even larger bHLH families, the functional characterization of their members lags behind, especially in wheat, in processes such as plant architecture establishment and biotic stress responses. This disparity highlights a critical knowledge gap. Bridging this gap is particularly important for maize and wheat, whose extensive cultivation and central role in global food security demand a deeper molecular understanding.

Despite the significant progress achieved, several key scientific questions demand further exploration to fully realize the potential of bHLH factors in cereal improvement:

**Molecular basis of functional redundancy in the bHLH family.** The bHLH family has a large number of members, and functional redundancy often occurs within the same subfamily or among different subfamilies [3], which complicates the study of their functions. Understanding the molecular basis of functional redundancy and function-specific regulation is an important challenge in current research. Future studies need to combine genetic, molecular biological, and bioinformatics methods to systematically analyze the interaction network among bHLH family members and the balance of functional redundancy and specific regulation.

**The elaborate regulatory network of bHLH proteins.** To date, functional analyses of bHLH transcription factors have mostly focused on a limited number of model genes and key physiological processes, while there remains a great deal of unknown territory regarding the functions of the entire family members. Particularly in maize and wheat, research on bHLH transcription factors in aspects such as plant architecture establishment and stress response is relatively lagging. As highlighted earlier, bridging this knowledge gap is a pressing task. Meanwhile, the precise mechanisms underlying the regulatory networks of bHLH transcription factors with known functions still require in-depth investigation, for example, their crosstalk with other signaling pathways, and the specific molecular mechanisms by which post-translational modifications modulate their functions. Addressing these questions will necessitate more detailed studies integrated with advanced technical approaches.

**Breeding application strategies of bHLH genes.** In-depth studies on the functions and regulatory mechanisms of bHLH transcription factors in cereal crops provide abundant gene resources and theoretical guidance for improving crop traits using molecular breeding technologies. Using gene editing technologies such as CRISPR/Cas9, the key bHLH genes can be modified to improve crop yield, quality, and stress resistance [138]. For example, editing rice *SD6* and its wheat homologous genes can reduce pre-harvest sprouting [77]. In addition, functional molecular markers can be developed based on natural variations in bHLH genes. For instance, natural variations in the promoter region of wheat *TabHLH27-A1* are associated with drought tolerance, yield and water use efficiency, which can be used as molecular markers for marker-assisted selection breeding [110]. The drought-tolerant allele of maize *ZmbHLH124* can also be used to develop molecular markers for screening drought-tolerant germplasm [106]. The use of these functional markers can accelerate the pyramid breeding process of important traits in cereal crops and improve breeding efficiency [139].

## Figures and Tables

**Table 4:** Characterized bHLH family genes regulating iron homeostasis.

Gene Name	Gene Accession	Function	Refs.
*OsIRO2/OsbHLH056*	Os01g0952800	Essential for iron acquisition	[87]
*OsFIT/OsbHLH156*	Os04g0381700	Promotes iron uptake	[89,90]
*OsIRO3*	Os03g0379300	Negatively regulate iron deficiency responses.	[92,93]
*OsbHLH060/OsPRI1*	Os08g0138500	Positively regulate iron deficiency responses.	[96]
*OsbHLH058/OsPRI2*	Os05g0455400		[94,95]
*OsbHLH059/OsPRI3*	Os02g0116600		[94,95]
*OsbHLH061*	Os11g0601700	Negatively regulates the long-distance transport of iron.	[97]
*OsbHLH133*	Os12g0508500	Regulate the distribution of iron between roots and shoots.	[98]
*ZmFIT*	Zm00001d025205	Promotes iron uptake	[99]
*ZmIRO2*	Zm00001d011847	Promotes iron uptake	[99]

## Data Availability

Data are available from the authors on request.
